# Multiple Drug-Resistant CLABSI from an Extremely Rare Bacterium, *Chryseobacterium gleum*

**DOI:** 10.1155/2020/2097813

**Published:** 2020-08-11

**Authors:** David Anson, Benjamin Chaucer, Joseph Norton, Saurabh Bansal

**Affiliations:** University of Illinois College of Medicine Peoria, Peoria, IL 61605, USA

## Abstract

*Chryseobacterium gleum* is a lactose nonfermenting Gram-negative bacillus (NFGNB) found in soil, plants, and some water sources but rarely implicated as a human pathogen. Its scarcity in the medical literature and resistance to numerous broad-spectrum antibiotics such as carbapenems, cephalosporins, and beta-lactam/lactamase inhibitors pose a diagnostic and therapeutic challenge. We present the first reported case, to the best of our knowledge, of sepsis from central line-associated blood stream infection from *Chryseobacterium gleum* in the United States.

## 1. Introduction

Healthcare-associated infections (HAIs) are one of the leading causes of morbidity and mortality in the United States and around the world, affecting hundreds of millions of patients each year [1] In the United States alone, HAIs involve more than 2 million patients annually, resulting in nearly 100,000 deaths and an average cost of 40 billion US dollars [2]. Among healthcare-associated infections, central line-associated blood stream infections (CLABSIs) are the most frequent cause, and most cases have been shown to be largely preventable through surveillance and infection-control practices [3].

Nonfermenting Gram-negative bacilli (NFGNB) are found in numerous reservoirs in the hospital setting and are notorious for their potential to colonize mechanical devices, resulting in CLABSI and ventilator-associated pneumonia (VAP). The vast majority of NFGNB respond to broad-spectrum antibiotic regimens, such as beta-lactam/lactamase inhibitor combinations, cefepime, aminoglycosides, or carbapenems [4]. *Chryseobacterium gleum*, a NFGNB and pathogen predominantly documented in southeast Asia, has been implicated in both CLABSI and VAP and is resistant to broad-spectrum antibiotics commonly used to treat NFGNB. We present the first reported case, to our knowledge, of sepsis from CLABSI due to *Chryseobacterium gleum* in the United States.

## 2. Case Description

A 76-year-old female with a past medical history of vascular dementia, stage 3 chronic kidney disease, hypertension, congestive heart failure, and *Clostridium difficile* toxic megacolon status post total colectomy with ileostomy presented with a 3-day history of fever, chills, dyspnea, and altered mentation. The patient had required multiple hospital admissions for severe dehydration due to high-output ileostomy loss despite loperamide and atropine-diphenoxylate therapy. She denied significant change in ileostomy output and had no recent travel or sick contacts. She had an indwelling central venous catheter and received 1 liter of lactated Ringer's solution daily for the last several months. She was admitted to the medical intermediate care unit, where she was found to be lethargic with a Glasgow Coma Scale score of 14. Physical exam was significant for coarse breath sounds bilaterally. Cardiovascular and abdominal exams were unremarkable. There was no erythema or edema noted around the central venous catheter site. She was febrile to 103.0°F, and serum creatinine was elevated at 1.50 mg/dL, albumin, 3.2 g/dL, and CRP, 2.88 mg/dL, with normal leukocyte count, lactic acid, troponin, and urinalysis. Chest radiograph revealed no acute cardiopulmonary process. Empiric antibiotic therapy with IV meropenem 1 g every 8 hours, IV vancomycin 30 mg/kg loading dose followed by 20 mg/kg every 12 hours, IV azithromycin 500 g daily, and aggressive IV rehydration was initiated. Two sets of blood cultures, one drawn peripherally and one from the central line, showed growth of bacteria identified on Gram stain as Gram-negative coccobacilli after 12 hours of growth. In the absence of any other focal infection, a diagnosis of CLABSI was made.

VITEK MS (bioMérieux) matrix-assisted laser desorption ionization time-of-flight mass spectrometry (MALDI-TOF) utilizing MYLA software v4.5.0-4 identified *Chryseobacterium gleum* in 3 of 4 blood cultures drawn 48 hours apart. VITEK 2 antimicrobial susceptibility testing, using CLSI guideline, revealed resistance to ampicillin, cefazolin, ceftriaxone, aztreonam, gentamicin, meropenem, and piperacillin/tazobactam. It was susceptible only to levofloxacin and TMP/SMX, as noted in Table 1. Intravenous levofloxacin 750 mg daily for 7 days was started. Transthoracic and transesophageal echocardiograms were negative for endocarditis. The patient's mental status and clinical condition improved significantly on IV levofloxacin. Serial blood cultures were drawn, and the central venous catheter was replaced after 48 hours of negative blood cultures. Sepsis resolved and she was stable for discharge after 7 days of IV levofloxacin. She was discharged on oral levofloxacin 750 mg daily for 7 days, for a total treatment duration of 14 days.

## 3. Discussion

*Chryseobacterium gleum* is a lactose nonfermenting Gram-negative bacillus that is dispersed in the community. Singhal et al. first described it in 1983 under the name *Flavobacterium gleum* [5]. Human clinical isolates from vaginal swabs were identified by Holmes et al. as early as 1984 [6]. In 1994, Vandamme and colleagues proposed the novel genus *Chryseobacterium* [7]. Despite its prevalence in soil, water, and plants, it is an uncommon human pathogen that has been rarely documented in the literature [8]. Most reported cases originate from India and southeastern Asia. It has been implicated in urinary tract infections, pneumonia, meningitis, endocarditis, and bacteremia (Ibid, 5). Reported risk factors for *Chryseobacterium* include immunosuppression, advanced age, prolonged hospitalization, indwelling medical devices, and broad-spectrum antibiotics (Ibid, 4). The SENTRY Antimicrobial Surveillance program, provided in the supplement, sponsored by JMI laboratories, is one of the longest-running programs monitoring worldwide pathogens and changes in antimicrobial resistance [9]. According to the SENTRY public dataset, from 2013–2017, *Chryseobacterium gleum* accounted for only 13 of the 151,572 infectious isolates worldwide. Interestingly, 11 isolates were identified from the United States and 10 of the 11 were from hospital-acquired pneumonia [10]. Although these isolates were noted in the SENTRY dataset, we were unable to find any further case reports documenting *C. gleum* in the United States. Specifically, there was no documented occurrence in the literature or SENTRY dataset of CLABSI-associated sepsis due to *C. gleum* in the United States. However, evidence suggests that VITEK MS does not optimally differentiate species of *Chryseobacterium,* particularly *C. gleum* [11]. Therefore, more accurate identification with 16S rRNA gene sequencing should be performed if local laboratory abilities allow [12].

From the literature, two reported cases of sepsis from *Chryseobacterium gleum* have been documented in India. Singhal et al. described a 64-year-old diabetic with chronic obstructive pulmonary disease and on chronic steroids who required intubation due to hospital-acquired pneumonia from *Pseudomonas aeruginosa* and was treated with imipenem. On treatment day 6, he developed sepsis from *Chryseobacterium gleum* [5]. Similarly, the patient presented by Jain et al. was mechanically ventilated and developed aspiration pneumonia with separate simultaneous isolates from tracheal aspirates and blood cultures positive for *Chryseobacterium gleum* [7]. Interestingly, while the patient in our study was never intubated, she did have a central venous catheter, was immunocompromised, and had multiple, recent prolonged hospitalizations. She did not have any recent travel or exposure to individuals who had recently traveled. Our patient also received broad-spectrum antibiotic therapy with a carbapenem, which may have contributed to selection for *Chryseobacterium gleum*. In our patient, just as in the other two, the strain responded to fluoroquinolone antibiotics. While the majority of cases have been susceptible to fluoroquinolones, the literature also includes *Chryseobacterium* with resistance to fluoroquinolones and sole susceptibility to tetracyclines [13].

## 4. Conclusion

Central line-associated blood stream infections are the most frequent cause of hospital-associated infection worldwide. While broad-spectrum antibiotics like carbapenems, beta-lactam/lactamase inhibitor combination, and cephalosporins are commonly used to treat sepsis and CLABSI, *Chryseobacterium gleum* susceptibility patterns demonstrate resistance to these antimicrobials. Due to extremely limited susceptibility, there is a potential for *C. gleum* to become a major infectious threat, especially in the immunocompromised population. *Chryseobacterium gleum* is not well known in the medical community and is very rarely documented, especially in the United States. Despite its scarcity, it is imperative for medical professionals to recognize potential risk factors and maintain clinical suspicion of *Chryseobacterium gleum* when appropriate. Adherence to hand hygiene, aseptic procedures, and prompt identification and treatment are all critical factors in preventing negative outcomes related to CLABSI or *Chryseobacterium gleum*.

## Figures and Tables

**Table 1:** Sensitivity of isolated *Chryseobacterium gleum* with concentrations.

Antibiotic	Concentration (mcg/ml)	VITEK 2 (CLSI)
Ampicillin	≥32	Resistant
Ampicillin/sulbactam	≥32	Resistant
Aztreonam	≥64	Resistant
Cefazolin	≥64	Resistant
Ceftriaxone	≥64	Resistant
Gentamicin	≥16	Resistant
Levofloxacin	0.25	Susceptible
Meropenem	≥16	Resistant
Piperacillin/tazobactam	≥128	Resistant
Trimeth/sulfamethoxazole	≤20	Susceptible

## Supplementary Materials

Supplementary Materials Activity of antimicrobial agents when tested against 14 *Chryseobacterium gleum* isolates in the SENTRY program collected from medical centers in North America and from medical centers in Canada and USA during 2013, 2014, 2015, 2016, 2017, and 2018.
